# Supplementary material for: P-TRAP: a Panicle Trait Phenotyping tool
Source: BMC Plant Biol. 2013 Aug 29;13:122. doi: 10.1186/1471-2229-13-122 (PMC3848748; doi:10.1186/1471-2229-13-122)
Supplement: Additional file 7 — P-TRAP source code. The Netbeans project that contains the source code of the application. [file 1471-2229-13-122-S7.zip › javadoc.zip!/javadoc/org-bioalg-reportprocessor/org/bioalg/reportprocessor/Report.html]

Report (ReportProcessor)


JavaScript is disabled on your browser.


- Package
- Class
- Tree
- Deprecated
- Index
- Help

*org.bioalg.reportprocessor 1.0*

- Prev Class
- Next Class

- Frames
- No Frames

- All Classes

- Summary:
- Nested |
- Field |
- Constr |
- Method

- Detail:
- Field |
- Constr |
- Method


org.bioalg.reportprocessor

## Class Report

- java.lang.Object
- - org.bioalg.reportprocessor.Report

- ---

    

  ```
  public class Report
  extends java.lang.Object
  ```

  The reports generator class.

  Author:
  :   faroq

- - ### Constructor Summary

    Constructors

    Constructor and Description

    `Report()`

    `Report(org.w3c.dom.Element gmXML,
    org.w3c.dom.Element pmXML)`

    Constructor by using xml files for the graph and particles models

    `Report(org.bioalg.graphprocessor.GraphModel gm)`

    `Report(org.bioalg.graphprocessor.GraphModel gm,
    org.biolag.particleprocessor.ParticlesModel pm)`

    `Report(org.biolag.particleprocessor.ParticlesModel pm)`
  - ### Method Summary

    Methods

    | Modifier and Type | Method and Description |
    | --- | --- |
    | `java.lang.String` | `generateReport()` Collects the results and generate a master report |
    | `java.lang.String` | `generateReport2()` Generate a detailed report of panicle and grain. |
    | `java.lang.String` | `generateReport3(java.lang.String prevrecord)` Generate a detailed report of the panicle and grains. |
    | `org.bioalg.graphprocessor.GraphModel` | `getGraphModel()` |
    | `java.io.File` | `getImagefile()` |
    | `double` | `getPanicleRadius(java.awt.Point center, int searchRadius)` Find the panicle diameter by using the Euclidian Distance Map within a search range centered at the first generating point (center parameter). |
    | `java.lang.String` | `GetParticlesReport()` Create and return a string report of the grain traits only. |
    | `org.biolag.particleprocessor.ParticlesModel` | `getPm()` |
    | `java.lang.String` | `GetSumParticlesReport()` Generate a summary report for the grains. |
    | `boolean` | `seperateJunctions()` |
    | `void` | `setGm(org.bioalg.graphprocessor.GraphModel gm)` |
    | `void` | `setImagefile(java.io.File imagefile)` |
    | `void` | `setPm(org.biolag.particleprocessor.ParticlesModel pm)` |

    - ### Methods inherited from class java.lang.Object

      `clone, equals, finalize, getClass, hashCode, notify, notifyAll, toString, wait, wait, wait`

- - ### Constructor Detail


    - #### Report

      ```
      public Report(org.bioalg.graphprocessor.GraphModel gm)
      ```


    - #### Report

      ```
      public Report()
      ```


    - #### Report

      ```
      public Report(org.biolag.particleprocessor.ParticlesModel pm)
      ```


    - #### Report

      ```
      public Report(org.bioalg.graphprocessor.GraphModel gm,
            org.biolag.particleprocessor.ParticlesModel pm)
      ```


    - #### Report

      ```
      public Report(org.w3c.dom.Element gmXML,
            org.w3c.dom.Element pmXML)
      ```

      Constructor by using xml files for the graph and particles models

      Parameters:
      :   `gmXML` - xml element of the graph stored in an xml file
      :   `pmXML` - xml element of the particles model stored in an xml file
  - ### Method Detail


    - #### getImagefile

      ```
      public java.io.File getImagefile()
      ```


    - #### setImagefile

      ```
      public void setImagefile(java.io.File imagefile)
      ```


    - #### generateReport

      ```
      public java.lang.String generateReport()
      ```

      Collects the results and generate a master report

      Returns:
      :   a string of that compose the report.


    - #### generateReport3

      ```
      public java.lang.String generateReport3(java.lang.String prevrecord)
      ```

      Generate a detailed report of the panicle and grains.

      Parameters:
      :   `prevrecord` - The panicle strucutre record which is then headed the grains' record.

      Returns:
      :   A string report.


    - #### getGraphModel

      ```
      public org.bioalg.graphprocessor.GraphModel getGraphModel()
      ```


    - #### setGm

      ```
      public void setGm(org.bioalg.graphprocessor.GraphModel gm)
      ```


    - #### getPm

      ```
      public org.biolag.particleprocessor.ParticlesModel getPm()
      ```


    - #### setPm

      ```
      public void setPm(org.biolag.particleprocessor.ParticlesModel pm)
      ```


    - #### generateReport2

      ```
      public java.lang.String generateReport2()
      ```

      Generate a detailed report of panicle and grain.

      Returns:


    - #### GetParticlesReport

      ```
      public java.lang.String GetParticlesReport()
      ```

      Create and return a string report of the grain traits only.


    - #### GetSumParticlesReport

      ```
      public java.lang.String GetSumParticlesReport()
      ```

      Generate a summary report for the grains.

      Returns:


    - #### seperateJunctions

      ```
      public boolean seperateJunctions()
      ```


    - #### getPanicleRadius

      ```
      public double getPanicleRadius(java.awt.Point center,
                            int searchRadius)
      ```

      Find the panicle diameter by using the Euclidian Distance Map within a
      search range centered at the first generating point (center parameter).
      The diameter is just the largest radius of the maximal disk inside the
      search

      Parameters:
      :   `center` - the point where the search has to be made
      :   `searchRadius` - the search radius centered at the center point

      Returns:
      :   the panicle radius


- Package
- Class
- Tree
- Deprecated
- Index
- Help

*org.bioalg.reportprocessor 1.0*

- Prev Class
- Next Class

- Frames
- No Frames

- All Classes

- Summary:
- Nested |
- Field |
- Constr |
- Method

- Detail:
- Field |
- Constr |
- Method
